# Bio-Based Aromatic Copolyesters: Influence of Chemical Microstructures on Thermal and Crystalline Properties

**DOI:** 10.3390/polym12040829

**Published:** 2020-04-05

**Authors:** Keling Hu

**Affiliations:** Key Laboratory of Functional Polymer Materials of MOE, College of Chemistry, Nankai University, Tianjin 300071, China; hukl@ntu.edu.sg

**Keywords:** bio-based, copolyester, chemical microstructures, thermal property, crystalline property

## Abstract

Aromatic copolyesters, derived from bio-based nipagin and eugenol, were synthesized with renewable 1,6-hexandiol as the spacer. Number-average, weight-average molecular weights (*M*_n_, *M*_w_), and polydispersity (*D*) values were determined by size exclusion chromatography (SEC). Chemical structures were confirmed by ^1^H NMR and ^13^C NMR spectroscopies. Chemical microstructure analysis suggested that the nipagin and eugenol-derived units were inserted into polymer chains in an arbitrary manner. Due to the short chain of 1,6-hexanediol, the splitting of magnetically different methylene carbons, adjacent to the alcohol-oxygens, proved to be more sensitive towards sequence distributions, at the dyed level, than those from 1,10-decanediol. Thermal gravimetric analysis (TGA) demonstrated that these polyester materials have excellent thermal stability (>360 °C), regardless of the content of eugenol-derived composition incorporated. Differential scanning calorimetric (DSC) and wide-angle X-ray diffraction (WXRD) experiments revealed the semicrystalline nature for this kind of copolyesters. The crystallinities gradually decreased with the increase of eugenol-derived composition. Thermal and crystalline properties were well discussed from the microscopic perspective. The point of this work lies in establishing guidance for future design and modification of high-performance polymer materials from the microscopic perspective.

## 1. Introduction

Mankind is facing severe challenges with the rapid development of economy and population growth. These challenges, include exhaustion of fossil resources, marine pollution, greenhouse effect, and so forth. All these problems urge people to adopt sustainable and green manner for social behaviors [1,2,3,4]. Bio-based polymer materials are attracting more and more attention from both academic and industrial aspects, especially for the synthesis and modification of bio-based degradable polymers (polyester, polyurethane, and polycarbonate) [5,6,7,8]. Recently our group has been dedicated to the study of bio-based polyester materials [9,10]. The initial monomer design and polymerization method have been used to improve the final property, however, strong demand still exists for the acquisition of high-performance bio-based polymeric materials [11,12,13,14].

Polymer properties depend on the chemical structure of material itself essentially [15,16]. For example, thermal and crystalline properties are closely related to structural units, and thus, are caused by different accumulations and motion of chain segments [17,18]. Functional groups on the main chains or side chains are critical for thermal stability. For example, heteroatoms (N, O, S) can easily degraded under high temperature or in the presence of oxygen [19,20,21,22]. Ester, urethane or carbonate groups are also easily attacked by other chemicals in the reaction systems, and thus, cause the scission of polymer chains [23,24,25,26]. Designing materials with specific functionality has advantages. These materials are stable during use, and are easily degraded under mild conditions, following use [27,28,29,30]. Consequently, the priorities involve the elegant design and derivatization of starting building blocks, and understanding how chemical structures influence the final properties, particularly from a microscopic view [31,32].

The compound, 4-Hydroxybenzoic acid, exists naturally in campanulaceae and ericaceous plants [33], and has been widely used as reactant in organic synthesis, especially for the synthesis of parabens. Nipagin, as the most widely studied paraben, can be used for the synthesis of various multifunctional polymeric materials, such as epoxy resins and liquid crystal materials [34,35]. Eugenol, extracted from clove oil [36], has been used for the synthesis of benzoxazines, coatings, etc [37,38]. Based on their availability and cheap price, they have promising uses in industrial applications, such as, food packing and beverage bottles. Recently, nipagin and eugenol are also used for the synthesis of polyester materials in our group. Further studies need to be performed, in order to better understanding the structure-property relationships.

In this study, starting from the renewable nipagin and eugenol, two kinds of diester monomers were synthesized via thiol-ene click chemistry and SN2 reactions [39,40]. Random copolymerization was then carried out in the predetermined composition ratios of diester monomers with another renewable monomer, 1,6-hexanediol, as a comonomer [41,42]. Sulphur atoms, phenoxy-links, and ester groups exist on the polymer main chains. This structural characteristic may have a critical influence on the final thermal and crystalline properties, which were studied and explained from the microscopic perspectives.

## 2. Experimental

### 2.1. Materials and Methods

The chemical reagents and materials, used in this project, and the general instrumentation and methods can be found in the supporting information section of this article. Scheme S1 (Supplementary Materials) illustrates the synthetic routes of N2, E1 and E2 monomers.

### 2.2. General Procedure for the Synthesis of Polyesters 

**Scheme 1:**
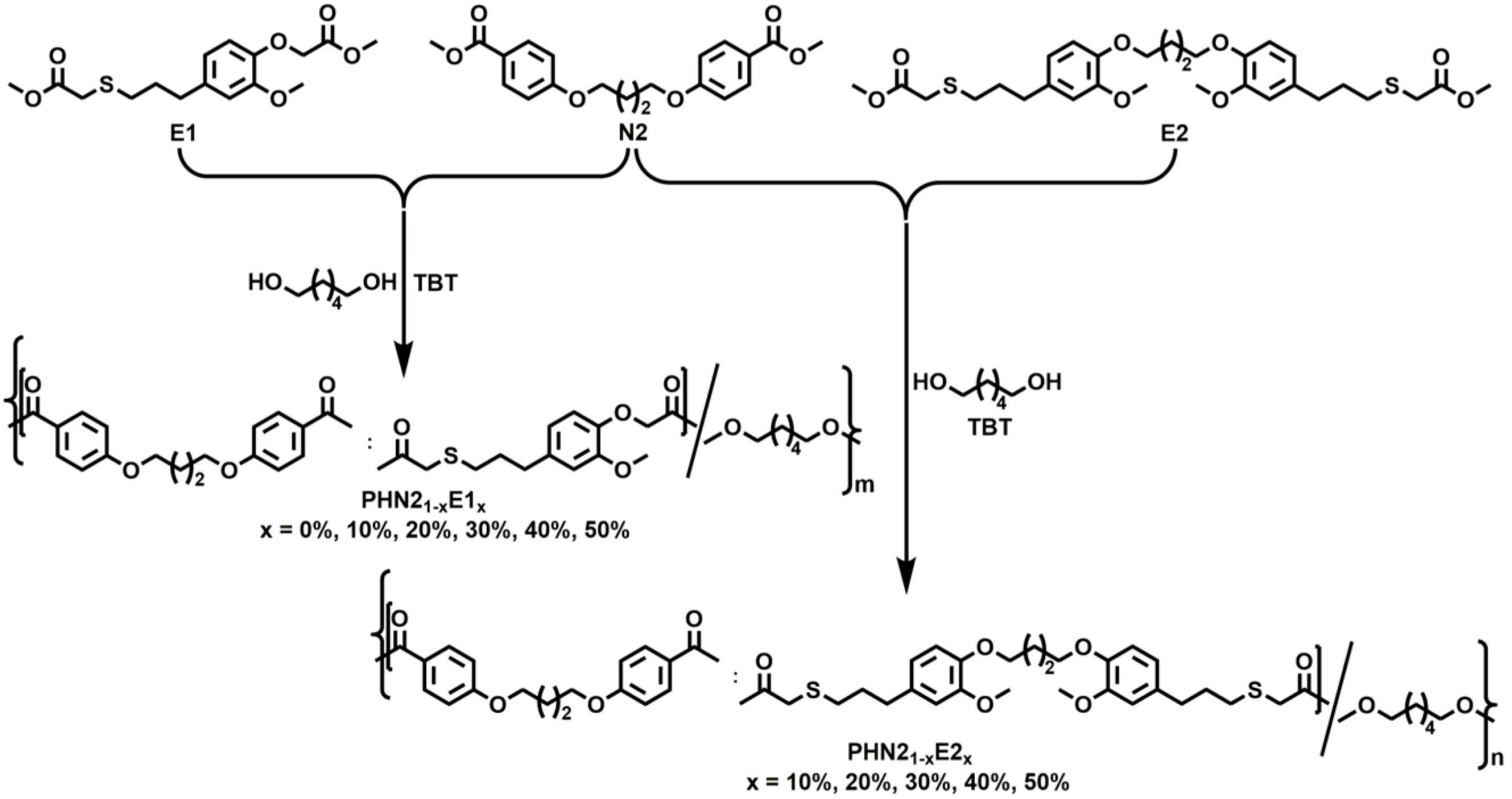
Synthetic pathway for the preparation of nipagin and eugenol-based PHN2_1−*x*_E1*_x_* and PHN2_1−*x*_E2*_x_*.

PHN2 homopolyester and PHN2_1−*x*_E1*_x_*, PHN2_1−*x*_E2*_x_* (x indicates molar ratio of E1 or E2 units, *x* = 10%, 20%, 30%, 40%, 50%) copolyesters were synthesized by melt polycondensation from a mixture of N2, E1 or E2, and 1,6-hexanediol with prescribed feed ratio. The reaction was performed in a 25 mL Schleck round-bottom flask equipped with a magnetic stirrer bar, a nitrogen inlet and a vacuum distillation outlet. The synthetic pathway is illustrated in Scheme 1. A slightly excess molar ratio of diol to diester (1.05:1) was chosen to guarantee the endcap of hydroxyl groups. Tetrabutyl titanate (TBT, 0.6% molar relative to diesters) was adopted as the catalyst. Before transesterification, the reaction system was purged with nitrogen for 15 min to ensure that no oxygen was left in the system, and to prevent the reaction mixture from being oxidized during polymerization. Transesterification was carried out at 140–180 °C for 3–5 h under a low nitrogen flow. Polycondensation was then carried out at 180–220 °C for 3–5 h under a 0.03–0.06 mbar vacuum until stirrer bar got stuck, suggesting the stop of polymerization. The pressure of reaction system was recovered to a normal level with nitrogen to prevent oxidation or degradation of product. The crude product was cooled to room temperature, dissolved in a minimum amount of chloroform and precipitated in an excess volume of methanol, in order to remove the oligomers and unreacted diols. The final product was obtained by suction filtration, washed with methanol, and dried in vacuum overnight.

**Figure 1:**
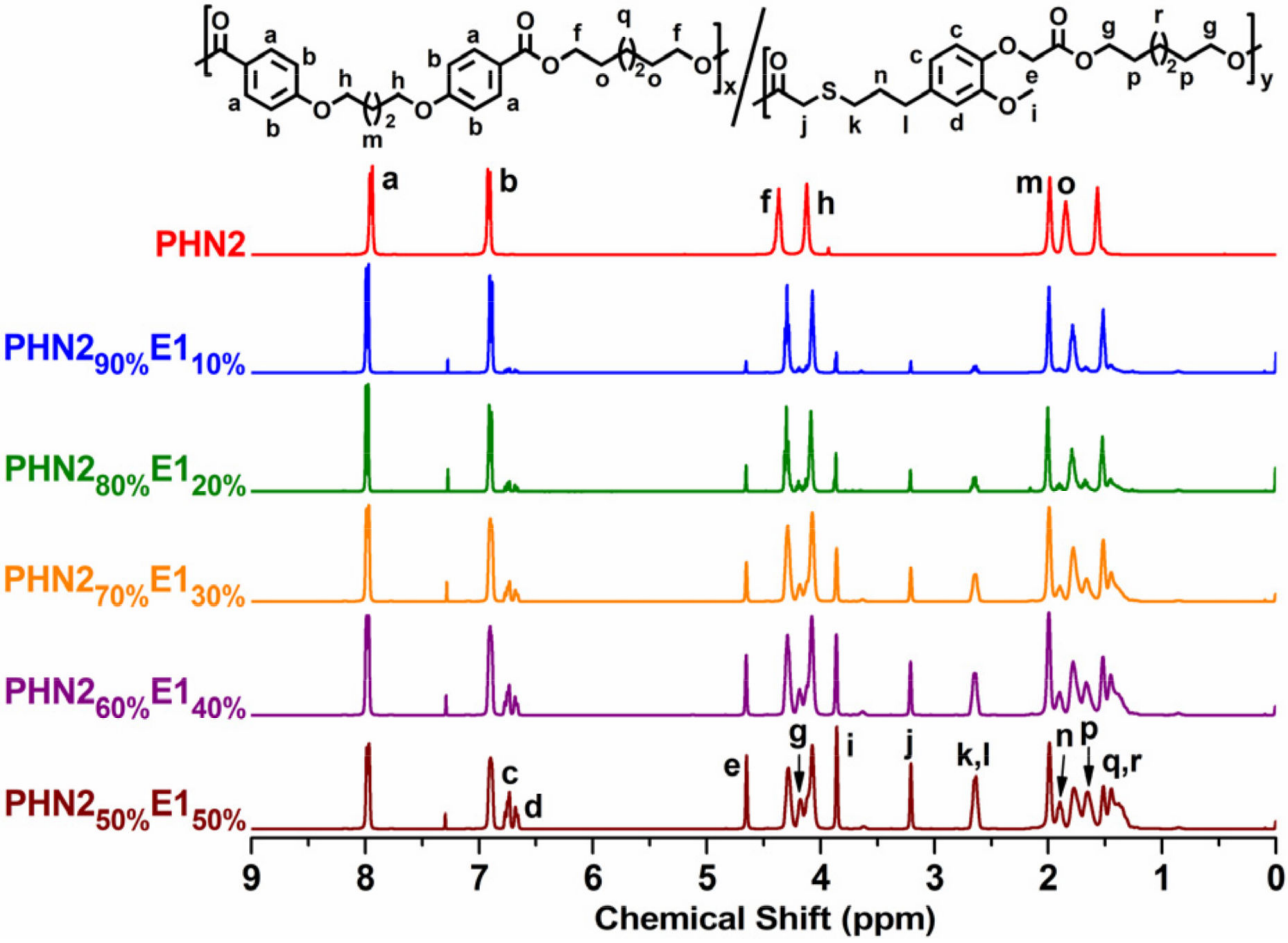
Compared ^1^H NMR spectra of PHN2_1−*x*_E1*_x_*.

**Figure 2:**
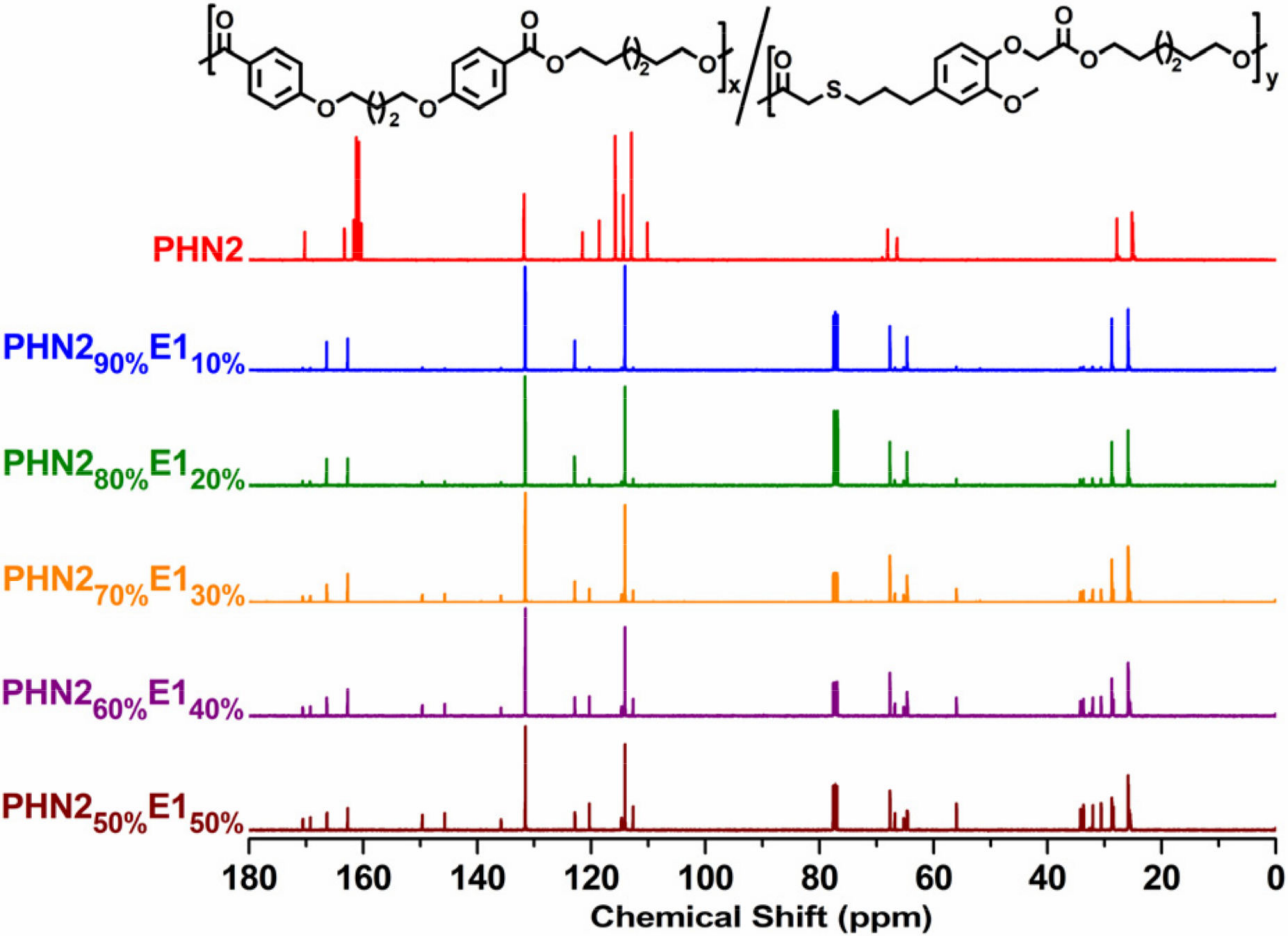
Compared ^13^C NMR spectra of PHN2_1−*x*_E1*_x_*.

The SEC traces are depicted in Figures S1 and S2 (Supplementary Materials). Chemical structures of the products were determined by ^1^H NMR (Figure 1 and Figure S3 in Supplementary Materials), and ^13^C NMR (Figure 2 and Figure S4 in Supplementary Materials). The peaks assignment was also given in the experimental section of supporting information. From the spectra, all the protons in chemical structures can find the corresponding signals.

## 3. Results and Discussion

### 3.1. Synthesis and Structures of the Polyesters

PHN2 homopolyester and PHN2_1−*x*_E1*_x_*, PHN2_1−*x*_E2*_x_* copolyesters were prepared by the melt polycondensation method in the predesigned composition ratios of nipagin and eugenol-derived dimethyl ester monomers with renewable 1,6-hexanediol as a comonomer. The polymerization pathway is illustrated in Scheme 1 and data are collected in Table 1. The symmetrical characteristic of N2 unit resulted in the high crystallizability of copolyesters. When the eugenol-derived components were below and equal 30% in the series of PHN2_1−*x*_E1*_x_*, these samples can’t dissolve in tetrahydrofuran (THF). Meanwhile, the samples for PHN2_1−*x*_E2*_x_* can dissolve in chloroform (CHCl_3_) only when eugenol-based components were below and equal 20%.

The high yields of products indicate the high reactivity and purity of these monomers. Nipagin and eugenol-derived units’ ratios in the final products of PHN2_1−*x*_E2*_x_* series matched very well with the feed ratios, indicating the excellent compatibility of the system during the whole polymerization process. However, for PHN2_1−*x*_E1*_x_* series, the units’ ratios in the final products gradually deviated from the feed ratios with the increase of eugenol-derived composition. This phenomenon may be ascribed to the high viscosity of the system at an early stage, resulting in the inadequate reaction of E1.

From the ^1^H NMR spectra of the two series of copolyesters (Figure 1 and Figure S3), the signal intensity corresponding to eugenol-derived units increases with the gradual insertion of eugenol-based composition, indicating the successful copolymerization of these two types of monomers.

**Figure 3:**
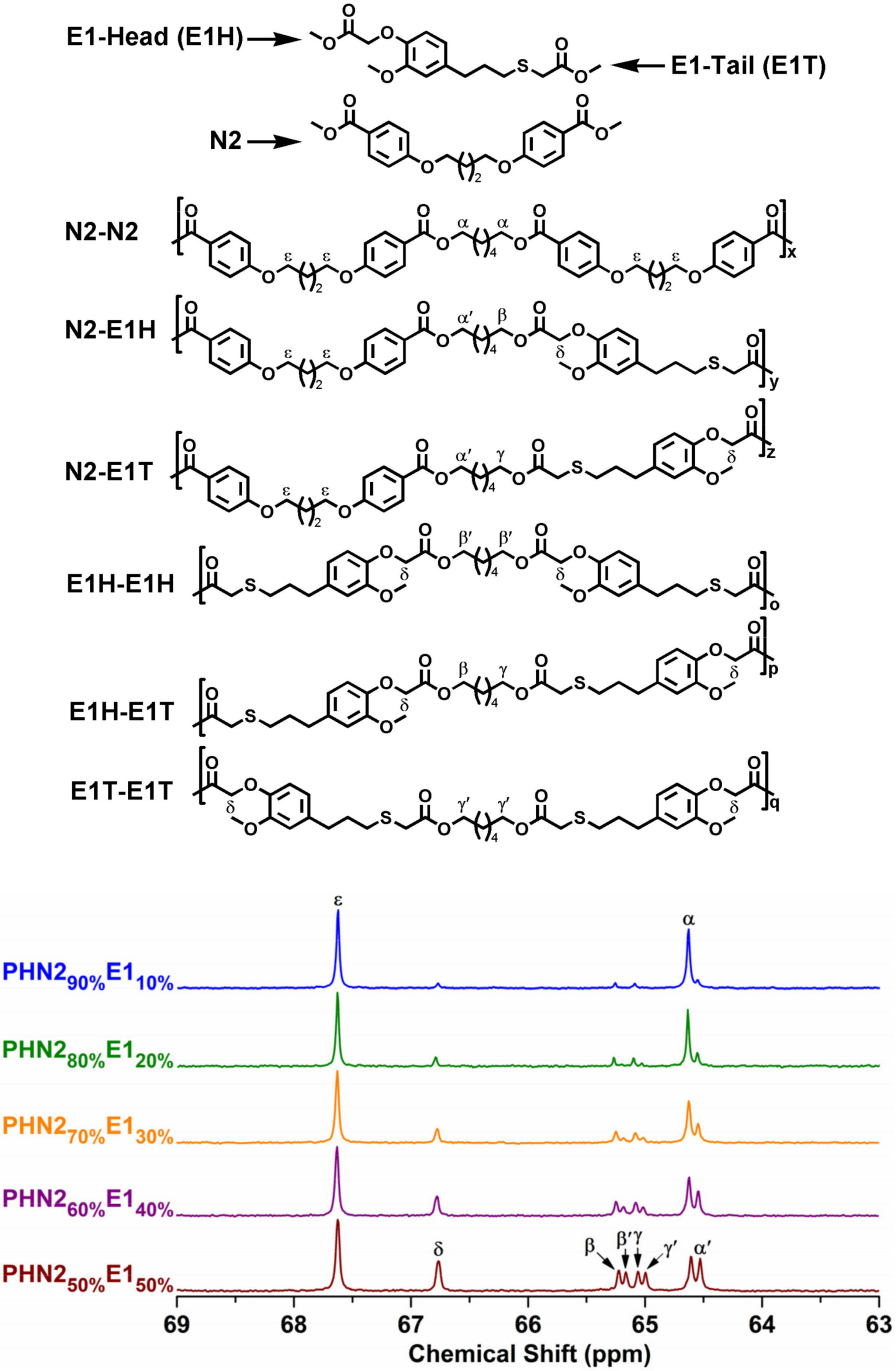
The splitting modes of the methylene carbons adjacent to the alcohol-oxygens for PHN2_1−*x*_E1*_x_* with the indications of the dyads to which they are assigned.

**Figure 4:**
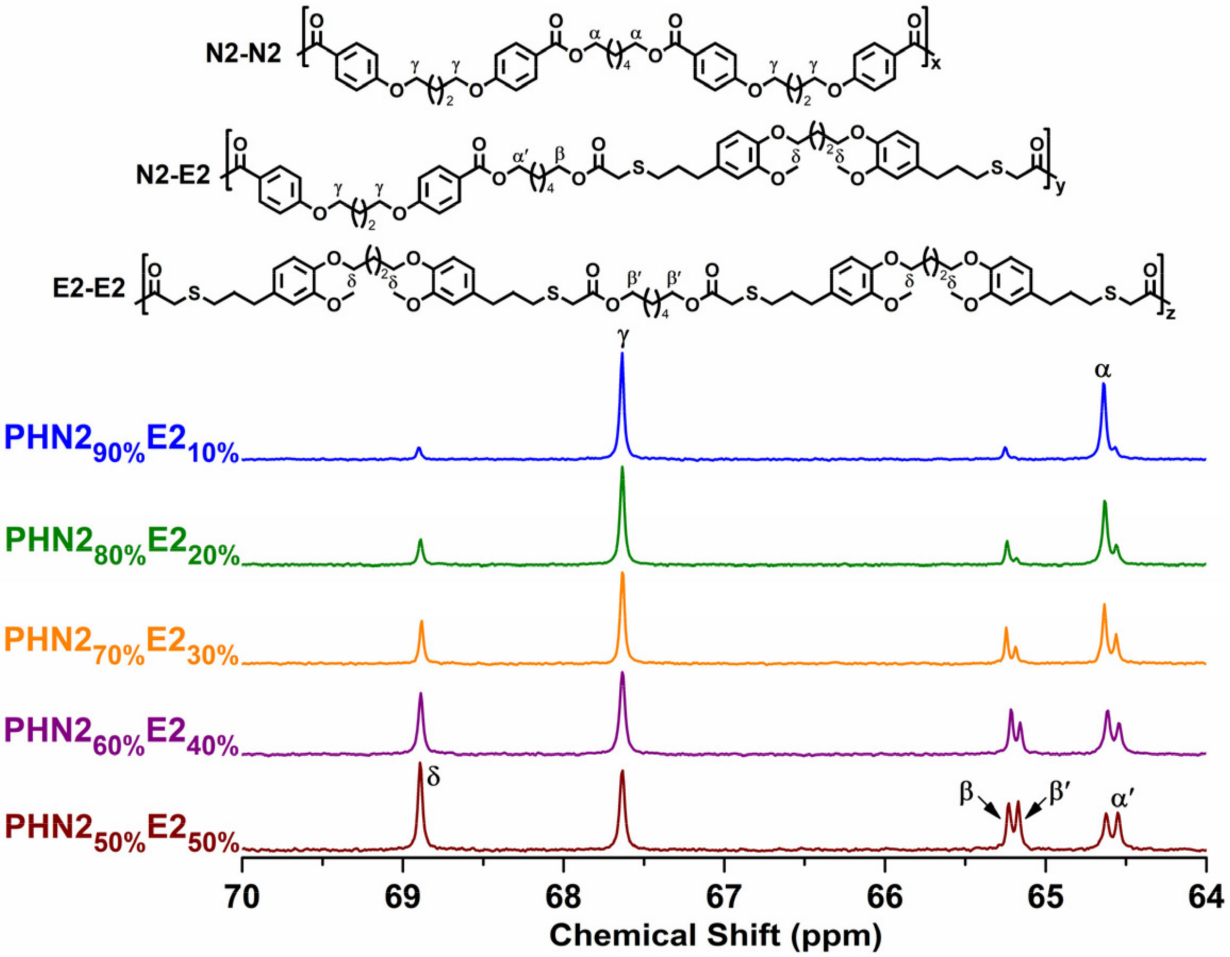
The splitting modes of the methylene carbons adjacent to the alcohol-oxygens for PHN2_1−*x*_E2*_x_* with the indications of the dyads to which they are assigned.

The chemical microstructures were studied by quantitative ^13^C NMR spectra thanks to the sensitiveness of magnetically different carbon atoms present in polyester backbones towards sequence distributions at the dyed level. [43,44] The possible sequence distributions and splitting modes of methylene carbons in ^13^C NMR spectra are depicted in Figure 3 and Figure 4, from which we can observe that the signal intensity closely related to the feed ratios. In this study, the methylene carbons connected to the alcohol-oxygen atoms proved to be well resolved in the ^13^C NMR spectra. All splitting modes of the peaks come from the different orientation of N2, E1, and E2 in polymer chains. However, the splitting mode of PHN2_1−*x*_E1*_x_* was more complicated than that of PHN2_1−*x*_E2*_x_* due to the asymmetrical feature of E1 unit, which generated a difference of *head* and *tail* featured by E1 unit when inserted into the polymer chains during copolymerizing. Meanwhile, N2 and E2 units did not have a *head* and *tail* difference because they are symmetrical. In the spectra of PHN2_1−*x*_E1*_x_*, *ε* signal originating from phenoxy-connected carbon in N2 featured a single peak and its signal intensity gradually decreased with the incorporation of eugenol-based composition. An opposite trend was observed for signal *δ*, which also exhibited a single peak in the whole series, indicating that signal *ε* and *δ* were not sensitive to sequence distributions. However, the methylene carbons connected to the alcohol-oxygens were very sensitive to sequence distributions, which split into three groups of signals *α* vs. *α’*, *β* vs. *β’* and *γ* vs. *γ’* (Figure 3). The signal intensities of *α’*, *β’* and *γ’* gradually increase compared with *α*, *β* and *γ*, respectively. Until the eugenol-based composition reached 50% the signal intensities in each group became almost the same. The opportunities of the six different sequence distributions were not equal when the amounts of nipagin and eugenol-based components were not the same. This phenomenon disappeared when feed ratio of the two diester monomers was 1:1.

As for PHN2_1−*x*_E2*_x_* series, another situation appeared as shown in Figure 4. Signal *δ* and *γ* also displayed single peaks as same as PHN2_1−*x*_E1*_x_*, but the methylene carbons connected to the alcohol-oxygen atoms split into only two groups of peaks (*α* vs. *α’* and *β* vs. *β’*) between 64–66 ppm. The reason for this was due to the same magnetic environment of the ester groups in respective N2 and E2 units. In each group, the peak intensities also grow from inequivalence to equivalence with the increase of eugenol-derived composition.

Furthermore, when compared with our previous report regarding the 1,10-decanediol derived copolyesters (Figures S5 and S6 in Supplementary Materials) [45], the splitting situation of methylene carbons adjacent to the alcohol-oxygens in PHN2_1−*x*_E1*_x_* and PHN2_1−*x*_E2*_x_* are more sensitive to sequence distributions because the chain length of 1,6-hexanediol is shorter than 1,10-decanediol. Consequently, the nipagin and eugenol-based units in polymer chains may have different constructions when the eugenol-based composition is the same. This difference may have critical influence on the final thermal and crystalline properties, which will be discussed in the following sections.

### 3.2. Thermal Properties

**Figure 5:**
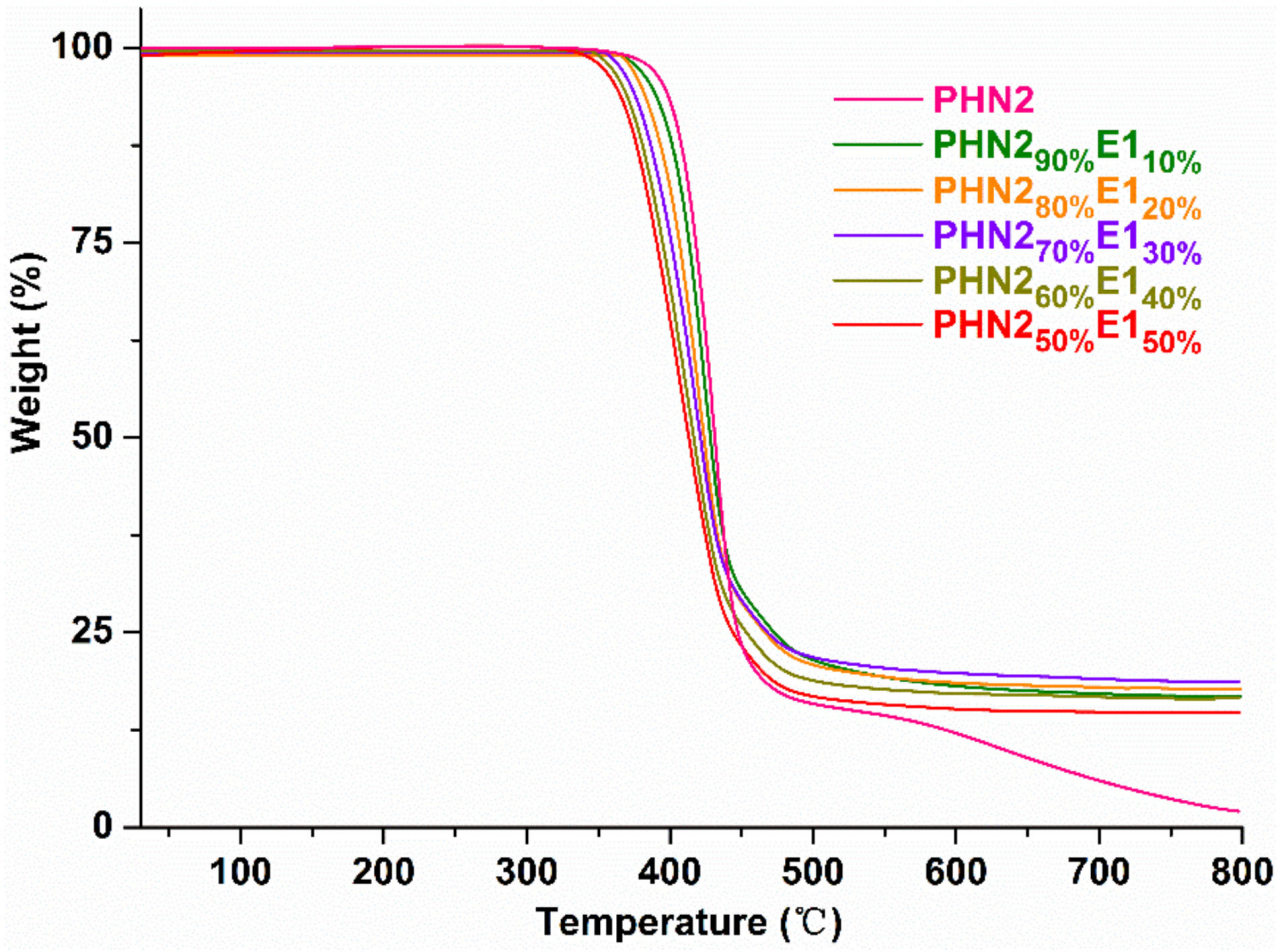
TGA curves of PHN2_1−*x*_E1*_x_* recorded from 25–800 °C at a heating rate of 10 °C min^−1^ under a nitrogen atmosphere.

**Figure 6:**
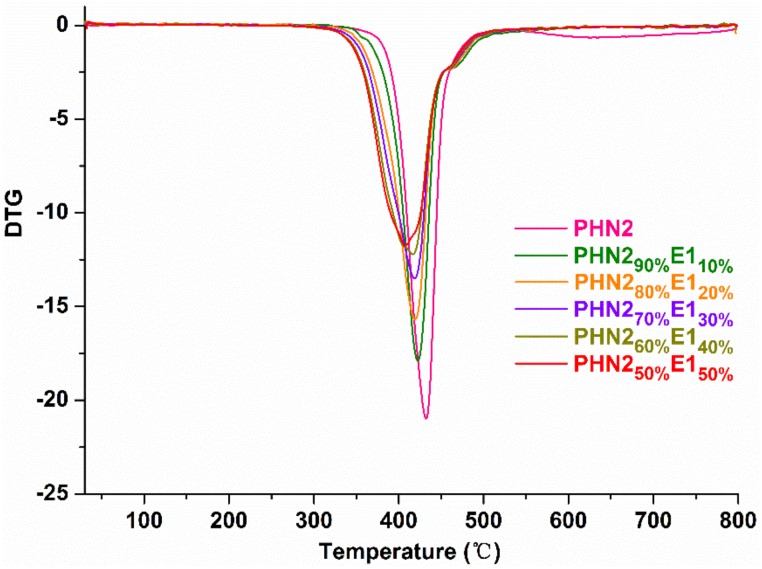
TGA derivative curves of PHN2_1−*x*_E1*_x_* recorded from 25–800 °C at a heating rate of 10 °C min^−1^ under a nitrogen atmosphere.

Thermal properties of the copolyesters were measured by thermogravimetric analysis (TGA). The weight-loss curves and corresponding derivative DTG curves are depicted in Figure 5 and Figure 6, and Figures S7 and S8 (Supplementary Materials), respectively, and the thermal parameters are gathered in Table 2. Thermal stability of PHN2_1−*x*_E1*_x_* and PHN2_1−*x*_E2*_x_* copolyesters gradually decreases with the increase of eugenol-based composition, compared with the parent PHN2 homopolyester. When the content of eugenol-derived composition reached 50%, the temperature at which 5% weight loss (*T*_5%_) decreased about 30 °C for PHN2_1−*x*_E1*_x_* and 20 °C for PHN2_1−*x*_E2*_x_* relative to PHN2. Furthermore, PHN2_1−*x*_E1*_x_* featured almost the same temperature for maximum degradation rate (*T*_d_) with PHN2_1−*x*_E2*_x_* when the eugenol-derived compositions were identical. From the above results, we conclude that the thermal stability of the final products gradually decreases with the incorporation of eugenol-derived composition. The *T*_5%_ values were still above 365 °C even though the content of eugenol-derived composition reached 50%. The *T*_d_ values were almost fixed at 420 °C when the eugenol-derived composition increased to 40% for PHN2_1−*x*_E1*_x_* series. The *T*_d_ value decreased about 10 °C when the eugenol-derived composition increased to 50% for PHN2_1−*x*_E1*_x_*. In the series of PHN2_1−*x*_E2*_x_*, we also see the same trend, that is, the *T*_d_ values were completely the same at 420 °C when the eugenol-derived composition below or equal to 30%. The *T*_d_ value decreased about 10 °C when the eugenol-derived composition exceeded 30%. From the microscopic view to explain this, the asymmetrical nature of E1 and E2 units and the existence of phenoxy and sulphur atoms both accelerate the degradation of samples. The asymmetrical structure of monomers leads to the loose packing of polymer chains and thus causes to be easily degraded under heat flow. Phenoxy and sulphur functional groups are all known sites to be attacked by impurities (O_2_) in the systems, resulting in the coloring of products. Consequently, *T*_5%_ and *T*_d_ values decrease with the eugenol-derived composition gradually increases.

Except for the parent PHN2 homopolyester, all the PHN2_1−*x*_E1*_x_* comopolyesters featured a two-step degradation mechanism. As for PHN2_1−*x*_E2*_x_*, PHN2_90%_E2_10%_ and PHN2_80%_E2_20%_ had a two-step degradation mechanism. However, single-step degradation was observed when the eugenol-derived composition was above 20%. This phenomenon can be explained by the mismatch of chain conformations at specific compositions [46,47]. When the eugenol-based composition was below (≤ 20%), asymmetrical eugenol-derived units were not averagely aligned along polymer chains. The nipagin-derived composition was the major and firstly degraded. Soon afterward, the second composition started to degrade with the increase of temperature. From the TGA data we can draw the conclusion that this kind of polyester materials have excellent thermal stabilities, and the eugenol-based composition has a critical influence on the final thermal stability from the microscopic perspective.

**Figure 7:**
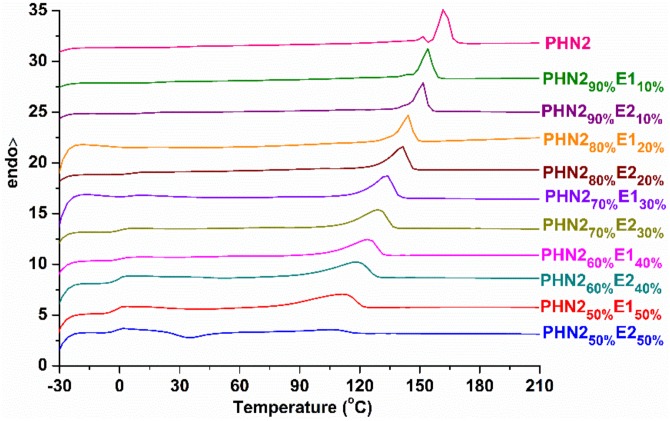
The second heating DSC curves for PHN2_1−x_E1_x_ and PHN2_1−*x*_E2*_x_* after precipitating from methanol carried out from −30 to 210 °C at a heating/cooling rate of 10 °C min^−1^.

Differential scanning calorimetric (DSC) analysis was used to study their other thermal and crystalline properties, like glass transition (*T*_g_), melting and crystallization temperatures (*T*_m_, *T*_c_), as well as their relevant melting and crystallization enthalpies (Δ*H*_m_, Δ*H*_c_), because these are critical factors to industry production and engineering applications [48,49]. The second DSC heating traces are depicted in Figure 7, and the corresponding thermal data are collected in Table 2. The full heating and cooling scans of DSC curves for PHN2_1−x_E1_x_ and PHN2_1−x_E2_x_ copolyesters are shown in Figures S9 and S10. *T*_g_s are found to become obvious with the gradual increase of eugenol-based composition because of the gradual increase of free volumes for segmental motion. In each series, *T*_g_ values gradually decrease with the insertion of eugenol-based composition. When the eugenol-based compositions were the same in the two series, PHN2_1−*x*_E1*_x_* has a slightly higher *T*_g_ value than PHN2_1−*x*_E2*_x_*, suggesting that E2 unit features a higher asymmetry than E1 unit. Except for the sample PHN2_50%_E2_50%_, all the other samples have the only melting peaks in the second heating runs. The Δ*H*_m_ values also gradually decrease with increase of eugenol-based composition in each series. More interestingly, PHN2 homopolyester and PHN2_90%_E1_10%_, PHN2_90%_E2_10%_ copolyesters have small melting peaks in front of the main melting peaks, indicating the second crystallization in the first cooling runs during DSC tests despite the relevant Δ*H*_m_ values are very small. However, PHN2_50%_E2_50%_ could not crystalize from the melt during the first cooling run (Figure S9 in Supplementary Materials). When the temperature reached about 34 °C, the conformation matching resulted in the start of crystallization. When temperature further increased, the melting peak appeared. In the microscopic level, the insertion of asymmetrical eugenol-based composition was unfavorable for the locally ordered packing of chain segments. The sequence distribution of *head*-*head*, *head*-*tail*, and *tail*-*tail* of nipagin and eugenol-derived units adopts an arbitrary manner along the polymer chains. Consequently, the variation trends of *T*_g_, *T*_m_, *T*_c_ and their corresponding Δ*H*_m_ and Δ*H*_c_ values can be explained reasonably.

### 3.3. Powder X-ray Diffraction Analysis

**Figure 8:**
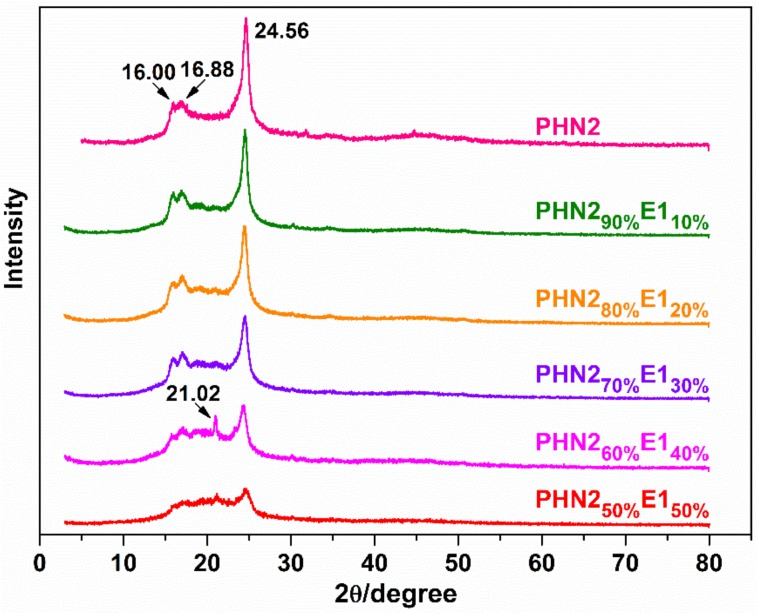
Powder WXRD profiles for PHN2_1−x_E1_x_.

Powder wide-angle X-ray diffraction analysis (WXRD) was carried out to supplement the DSC results. The WXRD curves are depicted in Figure 8 and Figure S11. From the traces, we observe that the polymer samples can form well discrete diffraction signals with the main peaks at about 24.5° characteristic of semi-crystalline materials. PHN2 homopolyester has a scattering pattern with four distinct reflections at 16.00°, 16.88°, 21.02° and 24.56°, respectively, which exactly correspond to the triclinic crystal structure just like displayed by PBT [50,51]. The diffraction pattern was reserved in the copolyesters, taking both diffraction angles and relative intensities into consideration, thereby suggesting that the crystalline manner of PHN2 could be retained in the copolyester samples. The crystallinity values calculated as the quotient between crystalline area and total area of the diffraction curves were given in Table 3. The crystallinities were found to decrease with the increase of eugenol-derived composition. The samples for WXRD were obtained directly from precipitation in methanol, which resulted in the slow alignment of polymer chains and certain degree of crystallization. Polymer chains became increasingly difficult to array orderly with the gradual increase of asymmetrical eugenol-based units. Consequently, the WXRD curves became increasingly smoother, particularly for the series of PHN2_1−*x*_E2*_x_*. Although, X-ray diffraction analysis can only qualitatively or semi-quantitatively allow the study the crystalline behavior, the consistency of DSC and WXRD results can provide good insight into the thermal and crystalline properties for this kind of polyester materials. The influence factors can be explained from the microscopic view, that is, chemical microstructures.

## 4. Conclusions

The correlations of thermal, crystalline properties with chemical microstructures were studied from the microscopic perspective, in order to gain a better understanding of the structure-property relationships of polymer materials. Renewable nipagin and eugenol-derived aromatic copolyester materials were firstly synthesized by the melt polycondensation method. The chemical structures and microstructures were confirmed by ^1^H NMR and ^13^C NMR. The effect of chemical microstructures on thermal and crystalline properties was emphatically investigated. When different amounts of asymmetrical eugenol-derived composition were incorporated, this kind of units were distributed in polymer chains evenly or not. Consequently, polymer chains became loose and easily degraded. DSC and WXRD results can also explain the crystalline properties from the microscopic perspective. With the insertion of asymmetrical eugenol-based composition, the locally ordered packing of chain segments could be easily destroyed. The sequence distribution of eugenol-derived units along the polymer chains leads to a decrease in crystallinity. The aim of this study is to provide a new perspective to study the influence factors for material properties and provide guidance for the future design and modification of polymer materials with high-performance.

## Figures and Tables

**Table 1:** Molar composition, molecular weight, polydispersity, isolated yield of the PHN2_1−x_E1_x_ and PHN2_1−*x*_E2*_x_*.

Copolyester	Yield (%)	Molar Composition					Molecular Weight		
		Feed Ratio			Final Product ^3^				
		*X*_N2_	*X*_E_		*X*_N2_	*X*_E_	*M*_n_	*M*_w_	*D*
PHN2 ^1^	86%	100	0		100	0	14200	24300	1.7
PHN2_90%_E1_10%_^1^	83%	90	10		89.3	10.7	18600	35900	1.9
PHN2_80%_E1_20%_^1^	85%	80	20		80.6	19.4	19200	37800	2.0
PHN2_70%_E1_30%_^1^	87%	70	30		70.8	29.2	20900	42100	2.0
PHN2_60%_E1_40%_^2^	81%	60	40		62.5	37.5	15100	28300	1.9
PHN2_50%_E1_50%_^2^	85%	50	50		53.2	46.8	19900	35400	1.8
PHN2_90%_E2_10%_^1^	82%	90	10		89.3	10.7	19800	39300	2.0
PHN2_80%_E2_20%_^1^	87%	80	20		80.0	20.0	20400	40200	2.0
PHN2_70%_E2_30%_^2^	86%	70	30		69.9	30.1	20400	37500	1.8
PHN2_60%_E2_40%_^2^	89%	60	40		59.9	40.1	14300	25900	1.8
PHN2_50%_E2_50%_^2^	88%	50	50		49.8	50.2	21000	37400	1.8

^1^ SEC carried out in CHCl_3_ against PS standards. ^2^ SEC carried out in THF against PS standards. ^3^ Molar composition of the final product determined by integration of the ^1^H NMR spectra.

**Table 2:** Thermal property parameters of PHN1_1−*x*_E1*_x_* and PHN1_1−*x*_E2*_x_*.

Polyester	TGA			DSC							
	*T*_5%_^1^ (°C)	*T*_d_^2^ (°C)	*W*^3^ (%)	First Cooling ^4^			Second Heating ^5^				
				*T*_c_ (°C)	*ΔH*_c_ (J g^−1^)		*T*_g_ (°C)	*T*_c_ (°C)	*ΔH*_c_ (J g^−1^)	*T*_m_ (°C)	Δ*H*_m_ (J g^−1^)
PHN2	395	432/614	2.1	131.0	163.8		32.2	-	-	*T*_m1_ = 152.4 *T*_m2_ = 160.3	Δ*H*_m1_ = 16.5 Δ*H*_m2_ = 147.8
PHN2_90%_E1_10%_	388	422	17.5	118.4	139.2		23.2	-	-	*T*_m1_ = 144.3 *T*_m2_ = 154.3	Δ*H*_m1_ = 8.3 Δ*H*_m2_ = 130.5
PHN2_80%_E1_20%_	381	419	18.6	105.9	109.2		12.5	-	-	144.3	108.7
PHN2_70%_E1_30%_	374	418	19.3	83.3	78.9		6.3	-	-	134.3	79.2
PHN2_60%_E1_40%_	367	417	17.0	63.3	54.3		0.5	-	-	124.2	54.6
PHN2_50%_E1_50%_	365	408	15.8	30.8	28.2		-5.8	-	-	111.7	28.5
PHN2_90%_E2_10%_	387	420	3.9	115.9	123.8		18.6	-	-	151.8	123.4
PHN2_80%_E2_20%_	384	420	5.3	98.4	98.7		6.4	-	-	141.8	99.2
PHN2_70%_E2_30%_	377	420	15.4	73.3	71.3		1.8	-	-	129.3	70.9
PHN2_60%_E2_40%_	378	412	13.4	35.8	49.1		-0.5	-	-	119.2	48.7
PHN2_50%_E2_50%_	376	410	12.1	-	-		-2.4	34.6	26.3	106.6	25.8

^1^ Temperature at which 5% weight loss. ^2^ Temperature for maximum degradation rate. ^3^ Remaining weight at 800 °C. ^4^ Crystallization temperatures (*T*_c_) and crystallization enthalpies (Δ*H*_c_) calculated from the first cooling DSC traces of the precipitated samples at a cooling rate of 10 °C min^−1^. ^5^ Glass transition, crystallization and melting temperatures (*T*_g_, *T*_c_, *T*_m_), melting and crystallization enthalpies (Δ*H*_m_, Δ*H*_c_) calculated from the second heating DSC traces of the precipitated samples at a heating rate of 10 °C min^-1^.

**Table 3:** Powder X-ray diffraction data for PHN2, PHN2_1−x_E1_x_, and PHN2_1−x_E2_x_.

Polyester	X-ray Diffraction Data				
	2θ (°) ^1^				*X*_c_^2^
**PHN2**	16.00 m	16.88 m	21.02 w	24.56 s	0.22
**PHN2_90%_E1_10%_**	16.00 m	16.88 m	21.02 w	24.56 s	0.19
**PHN2_80%_E1_20%_**	16.00 m	16.88 m	21.02 w	24.56 s	0.18
**PHN2_70%_E1_30%_**	16.00 m	16.88 m	21.02 w	24.56 s	0.16
**PHN2_60%_E1_40%_**	16.00 w	16.88 w	21.02 m	24.56 s	0.15
**PHN2_50%_E1_50%_**	16.00 w	16.88 w	21.02 w	24.56 m	0.18
**PHN2_90%_E2_10%_**	16.00 m	16.88 m	21.02 w	24.56 s	0.16
**PHN2_80%_E2_20%_**	16.00 m	16.88 m	21.02 w	24.56 s	0.15
**PHN2_70%_E2_30%_**	16.00 w	16.88 w	21.02 m	24.56 s	0.13
**PHN2_60%_E2_40%_**	16.00 w	16.88 w	21.02 w	24.56 m	0.11
**PHN2_50%_E2_50%_**	-	-	-	-	-

^1^ The diffraction angles measured in powder diffraction patterns for samples coming directly from precipitation in methanol and dried overnight. Intensities visually estimated as follows: m: medium; s: strong; w: weak. ^2^ Crystallinity indices calculated as the quotient between crystalline area and total area. Crystalline and amorphous areas in the X-ray diffraction pattern were quantified using PeakFit v4.12 software.

## Supplementary Materials

The following are available online at https://www.mdpi.com/2073-4360/12/4/829/s1, Chemical reagents and materials in experimental section used in this project. General instrumentation and methods. Synthesis of nipagin and eugenol-derived dimethyl esters. Scheme S1: Synthetic routes for the preparation of the nipagin and eugenol based dimethyl esters. Figure S1: SEC traces of polyester samples tested by CHCl_3_-phase GPC and the sample names are indicated in the figure. Figure S2: SEC traces of polyester samples tested by THF-phase GPC and the sample names are indicated in the figure. Figure S3: ^1^H NMR spectra of PHN2_1−x_E2_x_ copolyesters. Figure S4: ^13^C NMR spectra of PHN2_1−x_E2_x_ copolyesters. Figure S5: The splitting situations of the methylene carbons adjacent to the hydroxy-oxygens for PDN2_1−x_E1_x_ copolyesters with the indications of the dyads to which they are assigned. Figure S6: The splitting situations of the methylene carbons adjacent to the hydroxy-oxygens for PDN2_1−x_E2_x_ copolyesters with the indications of the dyads to which they are assigned. Figure S7: TGA curves of PHN2_1−x_E2_x_ copolyesters. Figure S8: TGA derivative curves of PHN2_1−x_E2_x_ copolyesters. Figure S9: Powder WXRD profiles for PHN2_1−x_E2_x_ copolyesters. Figure S10: The full heating and cooling scan of DSC curves of PHN2_1−x_E2_x_ after precipitating from methanol carried out from 30 to 210 °C at a heating/cooling rate of 10 °C min^−1^. Figure S11: Powder WXRD profiles for PHN2_1−x_E2_x_ copolyesters.
