# Supplementary material for: Bio-Based Aromatic Copolyesters: Influence of Chemical Microstructures on Thermal and Crystalline Properties
Source: Polymers (Basel). 2020 Apr 5;12(4):829. doi: 10.3390/polym12040829 (PMC7240576; doi:10.3390/polym12040829)
Supplement: Supplementary file 1 [file polymers-12-00829-s001.pdf]

Supporting Information for

Bio-based Aromatic Copolyesters: Influence of Chemical Microstructures on Thermal and Crystalline Properties

Keling Hu [1,2,*]

[1] Key Laboratory of Functional Polymer Materials of MOE, College of Chemistry, Nankai University, Tianjin 300071, China.

[2] *Current address:* Division of Chemistry and Biological Chemistry, School of Physical and Mathematical Sciences, Nanyang Technological University, 21 Nanyang Link, Singapore 637371, Singapore.

Correspondence to: *hukl@ntu.edu.sg*

1. Experimental Section

Chemical Reagents and Materials

Eugenol (99%, Sigma-Aldrich, St. Louis, USA), nipagin (99%, Sigma-Aldrich), methyl thioglycolate (99%, Sigma-Aldrich), methyl chloroacetate (98%, Sigma-Aldrich), 2,2-dimethoxy-2-phenylacetophenone (DMPA, 99%, Sigma-Aldrich), tetrabutyl titanate (TBT, 99.5%, Sigma-Aldrich), 1,6-hexanediol (98%, Aladdin, Shanghai, China), 1,4-dibromobutane (98%, Aladdin), potassium carbonate (K_2CO_3, 98%, Tianjin Chemical Reagent Corporation, Tianjin, China), potassium iodide (KI, 99%, Tianjin Chemical Reagent Corporation), acetonitrile (99.5%, Tianjin Chemical Reagent Corporation), chloroform ($CHCl_3$, 99.5%, Tianjin Chemical Reagent Corporation), tetrahydrofuran (THF, 99.5%, Tianjin Chemical Reagent Corporation), deuterated chloroform ($CDCl_3$, 99.8%, Qingdao Tenglong Weibo Technology. Co. Ltd., Qingdao, China), deuterated trifluoracetic acid (CF_3COOD, 99.5%, Qingdao Tenglong Weibo Technology), were used as received without further purification. Silica-gel slices used for thin-layer chromatography (TLC) were purchased from Qingdao Haiyang Chemical Co. Ltd., Qingdao, China.

General Instrumentation and Methods

^1H NMR and ^{13}C NMR spectra were recorded in $CDCl_3$ or CF_3COOD at 25 °C on a Bruker AVANCE III NMR spectrometer operating at 400 MHz and 100.6 MHz, respectively. Tetramethyl silane was used as the internal reference. Fourier transform infrared spectra (FTIR) were recorded on a Bio-Rad FTS6000 spectrophotometer at 25 °C. Polymer samples were prepared by grinding the polymeric materials adequately with potassium bromide powder, followed by compressing the mixture to form a pellet. Molecular weight and dispersity ($Đ$) of the materials were determined by size exclusion chromatography (SEC, Waters 2414 differential refraction detector) at 35 °C. THF or $CHCl_3$ was used as the eluent at a flow rate of 1.0 mL min^{-1}. The average molecular weights were calibrated against monodisperse polystyrene (PS) standards. Thermogravimetric analysis (TGA) was carried out using a NETZSCH TG209 instrument. In a typical method, polymer sample was heated from 25 to 800 °C under a nitrogen atmosphere at a rate of 10 °C min^{-1}. The temperature leading to 5% weight loss, the temperature for maximum degradation rate, and residue weight (%) at 800 °C were recorded. Differential scanning calorimetric (DSC) analysis was carried out using a Mettler-Toledo DSC Q100 calorimeter from TA Instruments. Polymer samples after precipitation from methanol were first heated from room temperature to 210 °C and hold at this temperature for 10 min to erase thermal history, then cooled to -30 °C. The glass transition temperatures (T_gs) were obtained from the second heating run. All runs were carried out at a rate of 10 °C min^{-1}. Indium was used as the calibration standards for temperature. Wide X-ray diffraction (WXRD) patterns were recorded on a D/max-2500 diffractometer using CuKα radiation with a wavelength of 0.1542 nm for powder samples after precipitation from methanol.

Synthesis of Nipagin and Eugenol based Dimethyl Esters

The preparation methods for nipagin and eugenol based dimethyl esters **N2**, **E1** and **E2** have been reported previously.[1,2] The synthetic routes of these monomers can also found in **Scheme S1**.

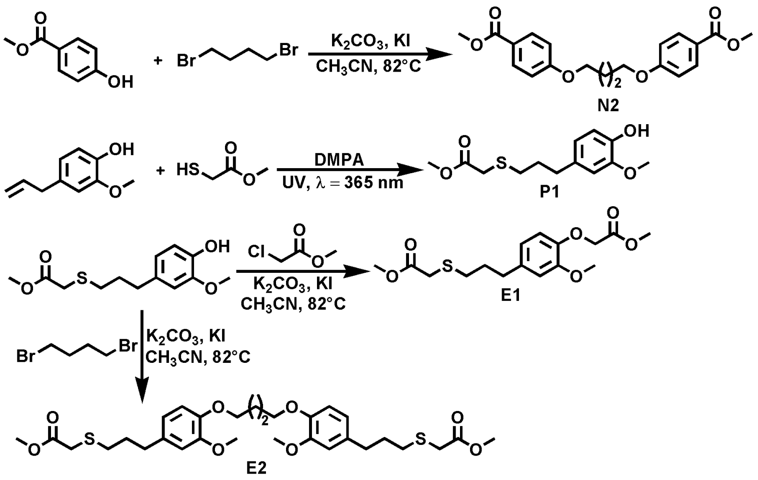

Scheme S1. Synthetic routes for the preparation of the nipagin and eugenol based dimethyl esters

2. Experimental Results

PHN2 Homopolyester. ^{1}H NMR (400 MHz, CF_3COOD, 25 °C, TMS): δ = 8.20-7.98 (m, 4·H, Ar-*H*), 7.16-6.94 (m, 4·H, Ar-*H*), 4.63-4.37 (m, 4·H, -COO-CH_2-), 4.36-4.11 (m, 4·H, ArO-CH_2-), 2.20-2.02 (m, 4·H, $ArOCH_2$-CH_2-), 2.02-1.85 (m, 4·H, -$COOCH_2$-CH_2-), 1.79-1.54 (m, 4·H, -$COOCH_2CH_2$-CH_2-) ppm; ^{13}C NMR (100.6 MHz, CF_3COOD, 25 °C, TMS): δ = 170.25 (Ar-*C*O-), 163.27 (Ar-*C*), 161.63 (solvent), 161.20 (solvent), 160.77 (solvent), 160.34 (solvent), 131.81 (Ar-*C*), 121.56 (Ar-*C*), 118.61 (Ar-*C*), 115.79 (solvent), 114.38 (Ar-*C*), 112.98 (solvent), 110.16 (Ar-*C*), 68.04 (ArO-*C*H_2-), 66.40 (-COO-*C*H_2-), 27.85 (-$COOCH_2$-*C*H_2-CH_2-), 25.19 (-$COOCH_2CH_2$-*C*H_2-), 25.05 ($ArOCH_2$-*C*H_2-) ppm.

PHN2$_{1-x}$E1$_x$ Copolyesters. ^{1}H NMR (400 MHz, $CDCl_3$, 25 °C, TMS): δ = 8.01-7.86 {m, (1-x)·4H; Ar-*H*}, 6.95-6.78 {m, (1-x) ·4H; Ar-*H*}, 6.77-6.67 (m, x·3H; Ar-*H*), 6.67-6.57 (m, x·H; Ar-*H*), 4.61 (s, x·2H; ArO-CH_2-CO-), 4.32-4.18 {m, (1-x)·4H; ArCOO-CH_2-}, 4.18-4.11 (m, x·4H; -COO-CH_2-), 4.11-3.93 {m, (1-x)·4H; ArO-CH_2-}, 3.82 (s, x·3H; ArO-CH_3), 3.17 (s, x·2H; -S-CH_2-CO-), 2.72-2.50 (m, x·4H; Ar-CH_2-CH_2-CH_2-S-), 2.06-1.91 {m, (1-x)·4H; $ArOCH_2$-CH_2-}, 1.91-1.81 (m, x·2H; $ArCH_2$-CH_2-CH_2S-), 1.80-1.67 {m, (1-x)·4H; $ArCOOCH_2$-CH_2-}, 1.67-1.53 (m, x·4H; -$COOCH_2$-CH_2-), 1.53-1.15 {m, (1-x)·4H+x·4H; -$COOCH_2CH_2$-CH_2-} ppm; ^{13}C NMR (100.6 MHz, $CDCl_3$, 25 °C, TMS): δ = 170.47 (-SCH_2-*C*O-), 169.17 ($ArOCH_2$-*C*O-), 166.26 (Ar-*C*O-), 162.61 (Ar-*C*), 149.55 (Ar-*C*), 145.59 (Ar-*C*), 135.72 (Ar-*C*), 131.47 (Ar-*C*), 122.79 (Ar-*C*), 120.24 (Ar-*C*), 114.62 (Ar-*C*), 114.00 (Ar-*C*), 112.59 (Ar-*C*), 67.54 (ArO-*C*H_2-CH_2-), 66.68 (ArO-*C*H_2-CO-), 65.14-64.44 (-COO-*C*H_2-), 55.87 (ArO-*C*H_3), 34.16 (-S-*C*H_2-CO-), 33.59 (Ar-*C*H_2-CH_2CH_2S-), 31.99 (-S-*C*H_2-CH_2CH_2Ar), 30.50 ($ArCH_2$-*C*H_2-SCH_2-), 28.64-28.34 (m, -$COOCH_2$-*C*H_2-), 25.77 ($ArOCH_2$-*C*H_2-), 25.62-25.32 (m, -$COOCH_2CH_2$-*C*H_2-) ppm.

PHN2$_{1-x}$E2$_x$ Copolyesters. ^{1}H NMR (400 MHz, $CDCl_3$, 25 °C, TMS): δ = 8.03-7.87 {m, (1-x)·4H; Ar-*H*}, 6.95-6.82 {m, (1-x)·4H; Ar-*H*}, 6.82-6.73 (m, x·H; Ar-*H*), 6.73-6.58 (m, x·2H; Ar-*H*), 4.37-4.18 {m, (1-x)·4H; -COO-CH_2-}, 4.17-3.93 {m, x·8H+(1-x) ·4H; ArO-CH_2-CH_2- and -COO-CH_2-}, 3.81 (s, x·6H; ArO-CH_3), 3.19 (s, x·4H; -S-CH_2-CO-), 2.75-2.53 (m, x·8H; Ar-CH_2-CH_2-CH_2-S-), 2.08-1.93 {m, (1-x) ·4H; $ArOCH_2$-CH_2-}, 1.93-1.82 (m, x·8H; $ArCH_2$-CH_2-CH_2S- and $ArOCH_2$-CH_2-), 1.82-1.69 {m, (1-x)·4H; $ArCOOCH_2$-CH_2-}, 1.69-1.56 (m, x·4H; -$COOCH_2$-CH_2-), 1.56-1.15 {m, (1-x)·4H+x·4H; -$COOCH_2CH_2$-CH_2-} ppm; ^{13}C NMR

(100.6 MHz, $CDCl_3$, 25 °C, TMS): δ = 170.51 (-SCH_2-*C*O-), 166.29 (Ar-*C*O-), 162.66 (Ar-*C*), 149.42 (Ar-*C*), 146.79 (Ar-*C*), 134.10 (Ar-*C*), 131.51 (Ar-*C*), 122.83 (Ar-*C*), 120.34 (Ar-*C*), 114.03 (Ar-*C*), 113.44 (Ar-*C*), 112.40 (Ar-*C*), 68.82 (ArO-*C*H_2-CH_2-), 67.56 (ArO-*C*H_2-CH_2-), 65.16-64.48 (-COO-*C*H_2-), 55.94 (ArO-*C*H_3), 34.18 (-S-*C*H_2-CO-), 33.64 (Ar-*C*H_2-CH_2CH_2S-), 32.05 ($ArCH_2CH_2$-*C*H_2-S-), 30.63 ($ArCH_2$-*C*H_2-CH_2S-), 28.67-28.40 (m, -$COOCH_2$-*C*H_2-), 26.04 ($ArOCH_2$-*C*H_2-), 25.81-25.43 (m, -$COOCH_2CH_2$-*C*H_2-) ppm.

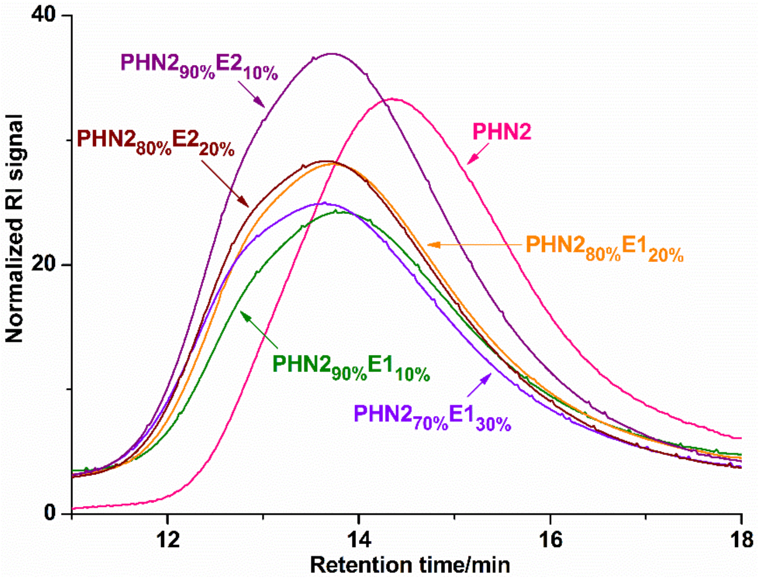

Figure S1. SEC traces of polyester samples tested by $CHCl_3$-phase SEC and the sample names are indicated in the figure.

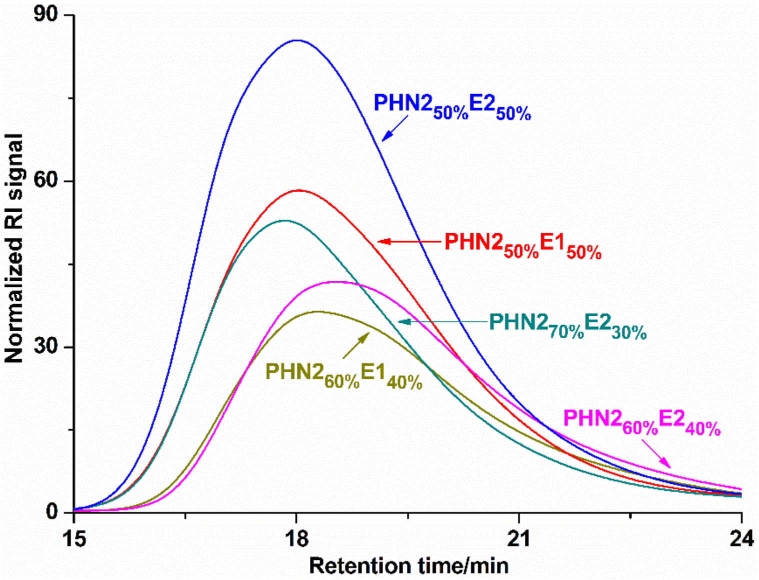

Figure S2. SEC traces of polyester samples tested by THF-phase SEC and the sample names are indicated in the figure.

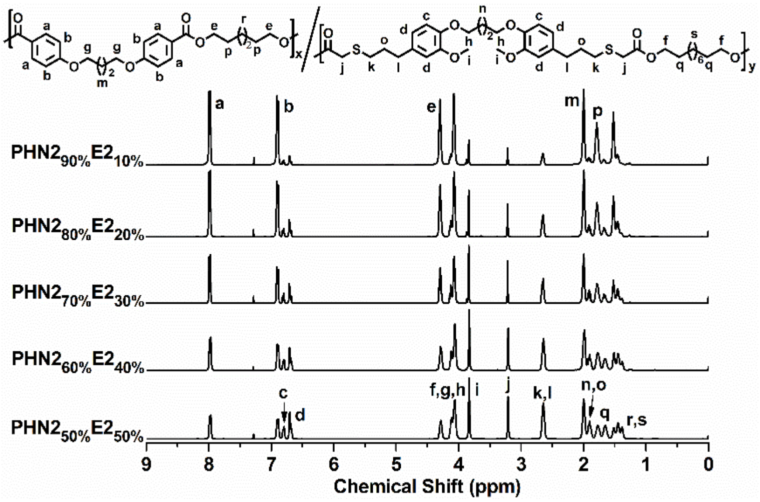

Figure S3. ^{1}H NMR spectra of PHN2$_{1-x}$E2$_x$ copolyesters.

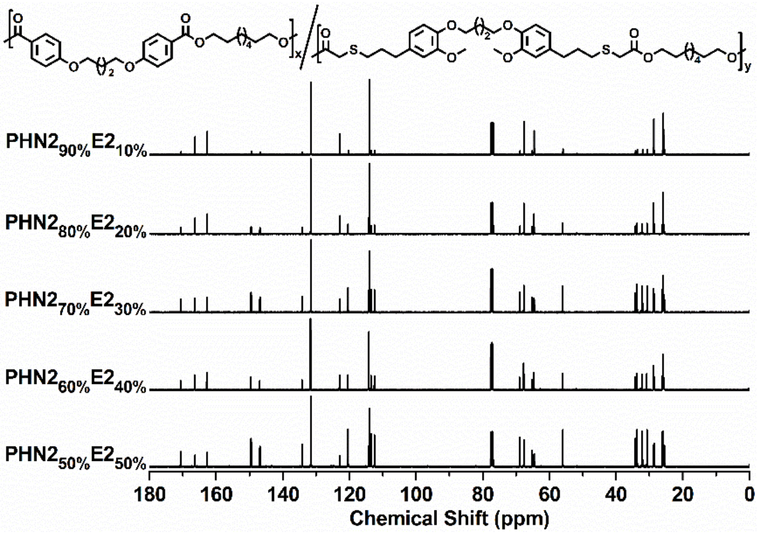

Figure S4. ^{13}C NMR spectra of PHN2$_{1-x}$E2$_x$ copolyesters.

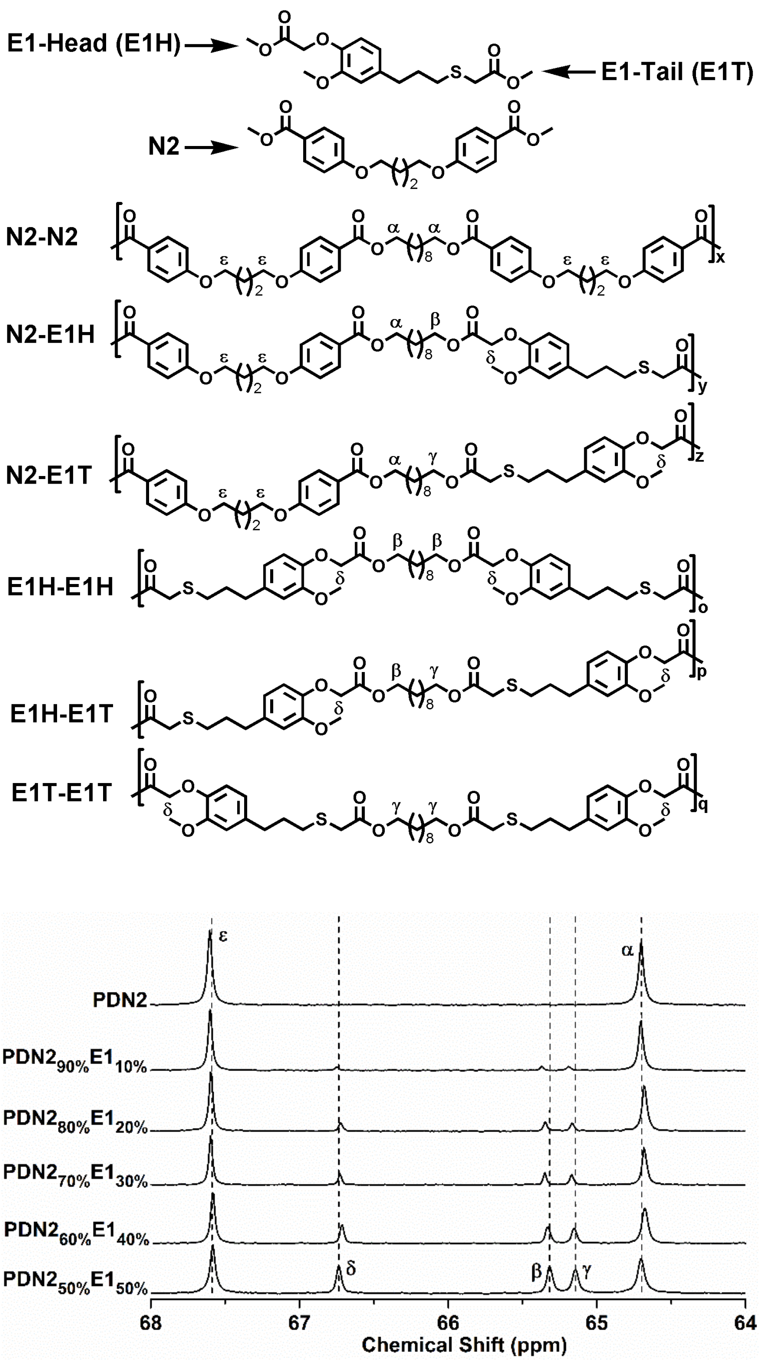

Figure S5. The splitting situations of the methylene carbons adjacent to the hydroxy-oxygens for $PDN2_{1-x}E1_x$ copolyesters with the indications of the dyads to which they are assigned.

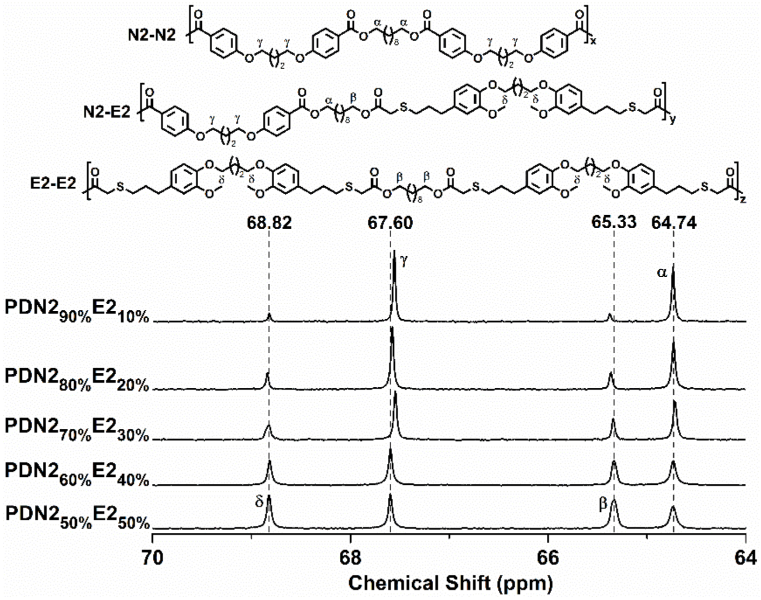

Figure S6. The splitting situations of the methylene carbons adjacent to the hydroxy-oxygens for PDN2$_{1-x}$E2$_{x}$ copolyesters with the indications of the dyads to which they are assigned.

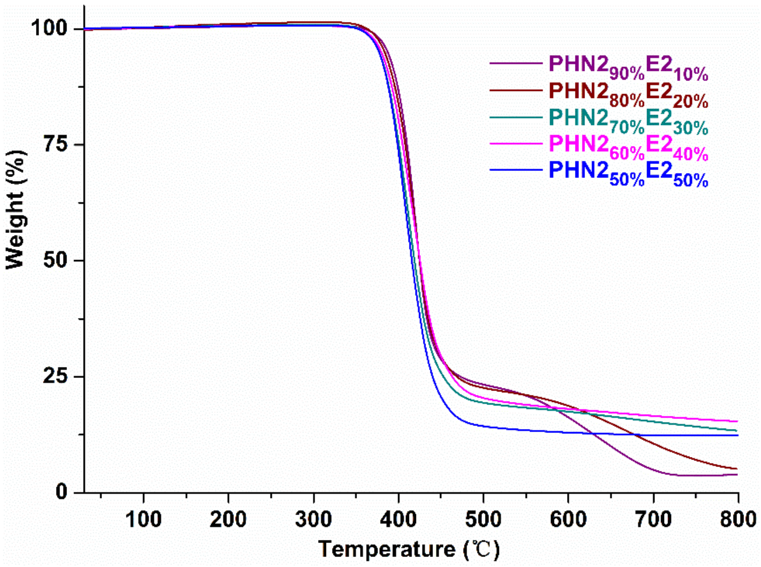

Figure S7. TGA curves of PHN2$_{1-x}$E2$_x$ copolyesters.

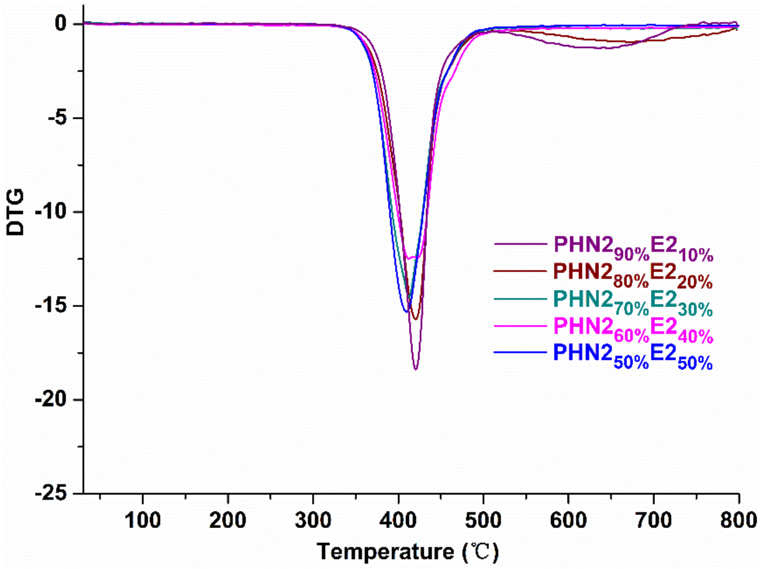

Figure S8. TGA derivative curves of PHN2$_{1-x}$E2$_x$ copolyesters.

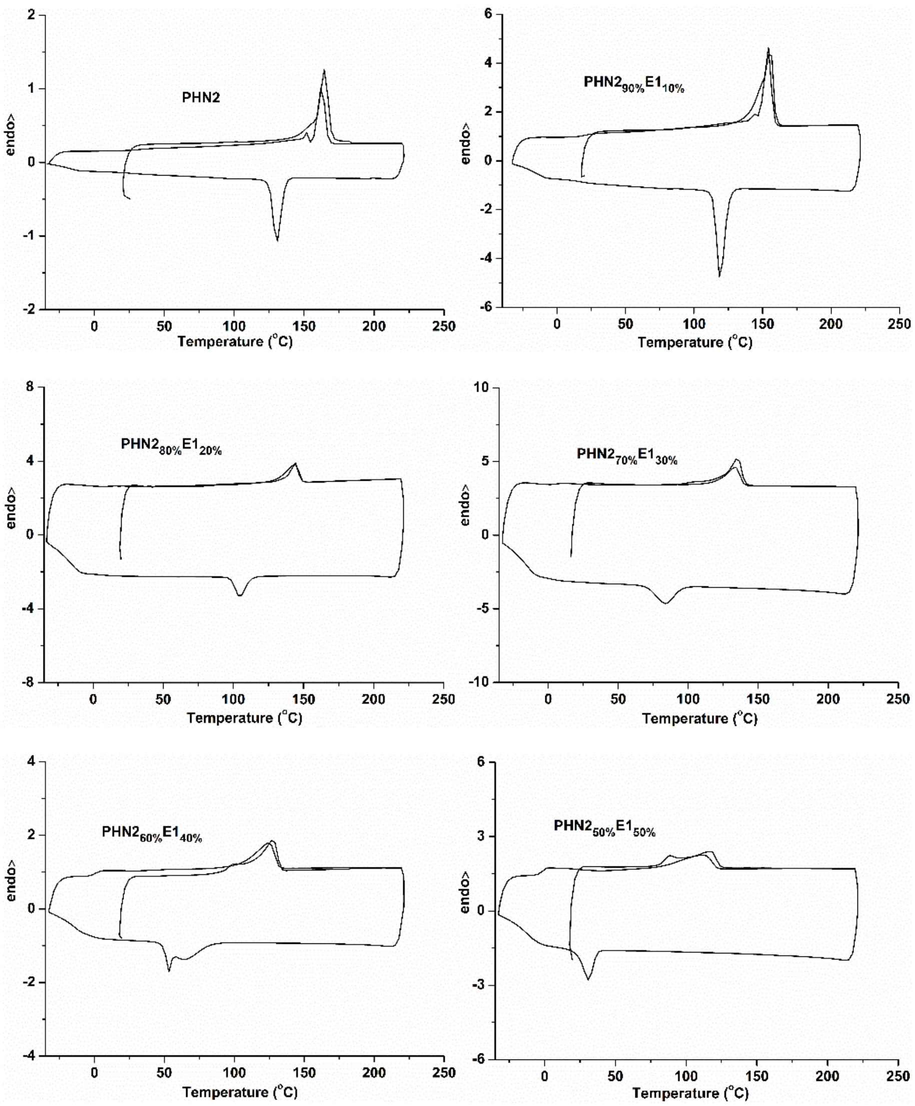

Figure S9. The full heating and cooling scan of DSC curves of PHN2 and PHN2$_{1-x}$E1$_x$ after precipitating from methanol carried out from -30 to 210 °C at a heating/cooling rate of 10 °C min^{-1}.

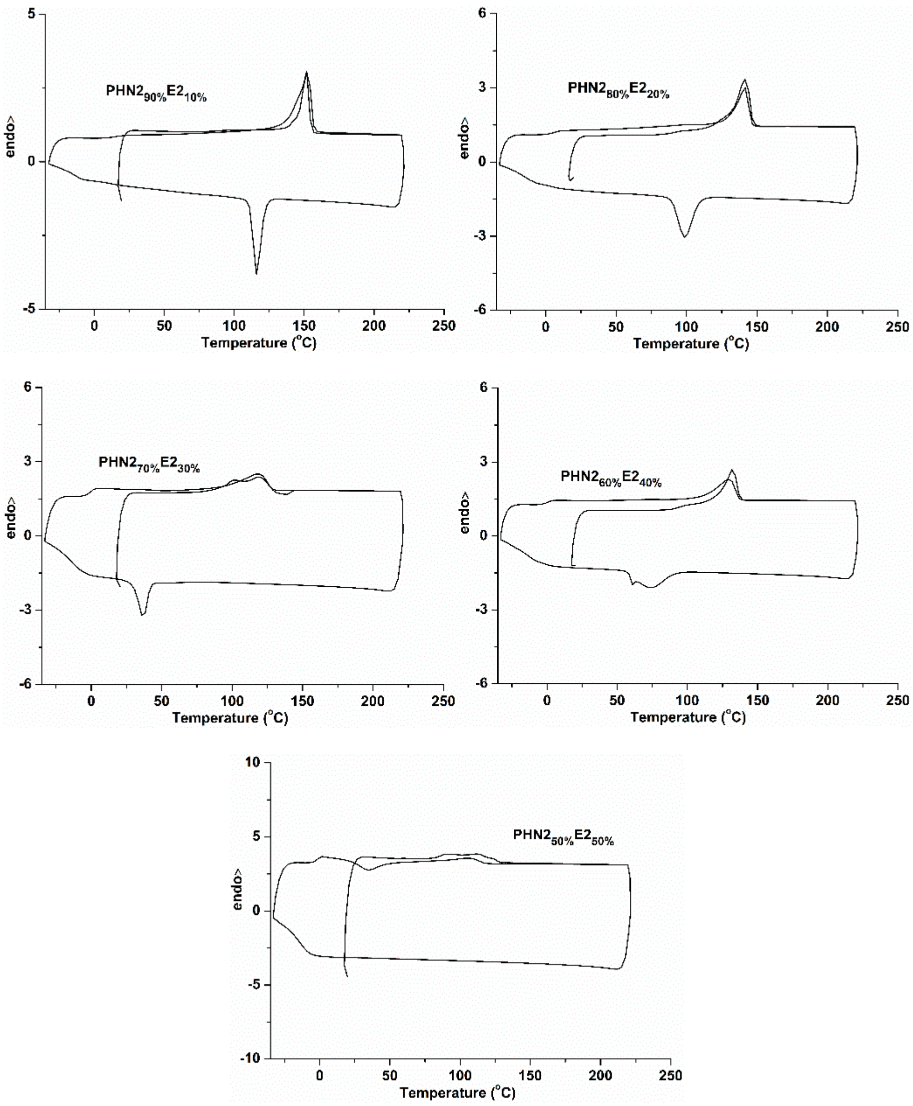

Figure S10. The full heating and cooling scan of DSC curves of PHN2$_{1-x}$E2$_{x}$ after precipitating from methanol carried out from -30 to 210 °C at a heating/cooling rate of 10 °C min^{-1}.

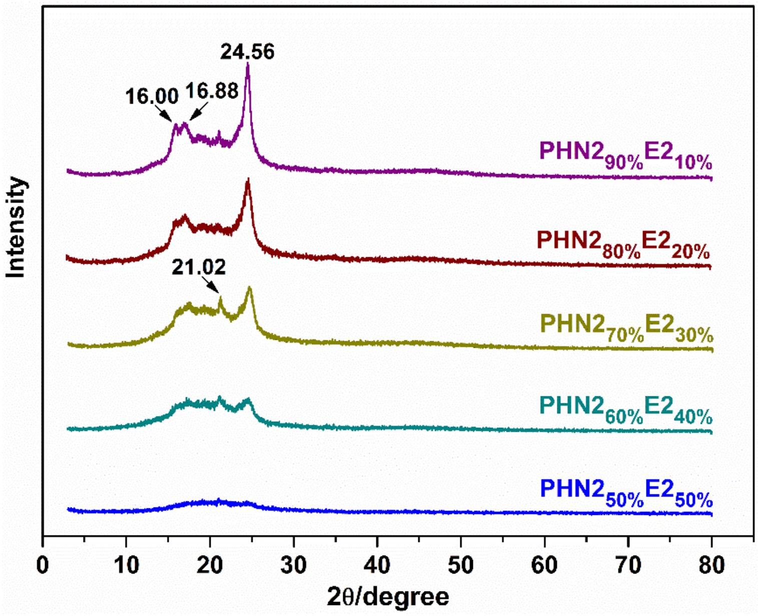

Figure S11. Powder WXRD profiles for PHN2$_{1-x}$E2$_x$ copolyesters.

3. References

1. K. L. Hu, D. P. Zhao, G. L. Wu and J. B. Ma, *Polym. Chem.*, 2015, **6**, 7138–7148.

2. K. L. Hu, D. P. Zhao, G. L. Wu and J. B. Ma, *J. Polym. Sci., Part A: Polym. Chem.*, 2016, **54**, 2171–2183.
